# Establishment of a multi-parameters MRI model for predicting small lymph nodes metastases (<10 mm) in patients with resected pancreatic ductal adenocarcinoma

**DOI:** 10.1007/s00261-021-03347-7

**Published:** 2021-11-20

**Authors:** Yan-Jie Shi, Bo-Nan Liu, Xiao-Ting Li, Hai-Tao Zhu, Yi-Yuan Wei, Bo Zhao, Shao-Shuai Sun, Ying-Shi Sun, Chun-Yi Hao

**Affiliations:** 1 Key Laboratory of Carcinogenesis and Translational Research (Ministry of Education), Department of Radiology, Peking University Cancer Hospital & Institute, No.52 Fu Cheng Road, Hai Dian District, Beijing, 100142 China; 2 Key Laboratory of Carcinogenesis and Translational Research (Ministry of Education), Department of Hepato-Pancreato-Biliary Surgery, Peking University Cancer Hospital & Institute, No.52 Fu Cheng Road, Hai Dian District, Beijing, 100142 China

**Keywords:** Pancreatic ductal carcinoma, Magnetic resonance imaging, Diffusion-weighted imaging, Lymph nodes

## Abstract

**Purpose:**

To evaluate the potential role of MR findings and DWI parameters in predicting small regional lymph nodes metastases (with short-axis diameter < 10 mm) in pancreatic ductal adenocarcinomas (PDACs).

**Methods:**

A total of 127 patients, 82 in training group and 45 in testing group, with histopathologically diagnosed PDACs who underwent pancreatectomy were retrospectively analyzed. PDACs were divided into two groups of positive and negative lymph node metastases (LNM) based on the pathological results. Pancreatic cancer characteristics, short axis of largest lymph node, and DWI parameters of PDACs were evaluated.

**Results:**

Univariate and multivariate analyses showed that extrapancreatic distance of tumor invasion, short-axis diameter of the largest lymph node, and mean diffusivity of tumor were independently associated with small LNM in patients with PDACs. The combining MRI diagnostic model yielded AUCs of 0.836 and 0.873, and accuracies of 81.7% and 80% in the training and testing groups. The AUC of the MRI model for predicting LNM was higher than that of subjective MRI diagnosis in the training group (rater 1, *P* = 0.01; rater 2, 0.008) and in a testing group (rater 1, *P* = 0.036; rater 2, 0.024). Comparing the subjective diagnosis, the error rate of the MRI model was decreased. The defined LNM-positive group by the MRI model showed significantly inferior overall survival compared to the negative group (*P* = 0.006).

**Conclusions:**

The MRI model showed excellent performance for individualized and noninvasive prediction of small regional LNM in PDACs. It may be used to identify PDACs with small LNM and contribute to determining an appropriate treatment strategy for PDACs.

## Introduction

Pancreatic ductal adenocarcinoma (PDAC) is the common malignant tumor in pancreas and the fourth leading cause of cancer-related death [1]. The prognosis of patients with PDAC remains poor, with a 5-year survival rate of only 9% [1]. At the time of diagnosis, 15–20% of patients have a potentially resectable disease with a 5-year survival rate of 10–20% [2]. Lymph node metastasis (LNM) has been regarded as a definite poor prognostic factor affecting early recurrence of the tumor and poor survival for patients with PDAC after surgery [3, 4]. In addition, a previous study showed that adjuvant chemoradiotherapy in PDAC was related to a significant improvement of survival only in patients with LN-positive disease, while the effects of chemoradiotherapy for a patient with N0 may be limited [5]. Therefore, the identification of reliable predictors of LNM in PDAC is of important significance for clinical decision-making.

In general, imaging criteria for metastatic lymph nodes include short-axis diameter that is more than 10 mm in size, irregular margin, and central necrosis based on imaging [6]. Still, in resectable PDAC, conventional imaging methods, such as computed tomography (CT), magnetic resonance imaging (MRI), positron emission tomography, and endoscopic ultrasound, are not accurate for the prediction of nodal metastases [7, 8]. The major reason is that a small lymph node with a short-axis diameter of less than 10 mm can also be metastatic in PDAC [9, 10]. Consequently, preoperative diagnosis of small lymph node metastases (less than 10 mm) remains a challenge.

MRI with diffusion-weighted imaging (DWI) can be used to noninvasively assess the pancreatic tumor, neighboring soft tissues, microvascular invasion, water diffusion behavior, and LNM in one examination [11]. Several studies have reported positive results for identifying metastatic lymph nodes in pancreatic tumors through the use of MRI findings, apparent diffusion coefficient (ADC), and intravoxel incoherent motion (IVIM) [11–13]. It is possible that MRI with DWI might show differences in morphology, water diffusion, heterogeneity, and microenvironment characteristics among metastatic and non-metastatic for small lymph nodes. Consequently, the purpose of this study was to evaluate the potential role of conventional MR findings, combined with DWI, in discriminating between metastatic and non-metastatic for small lymph nodes (<10 mm) in PDACs.

## Materials and methods

### Patients

**Fig. 1:**
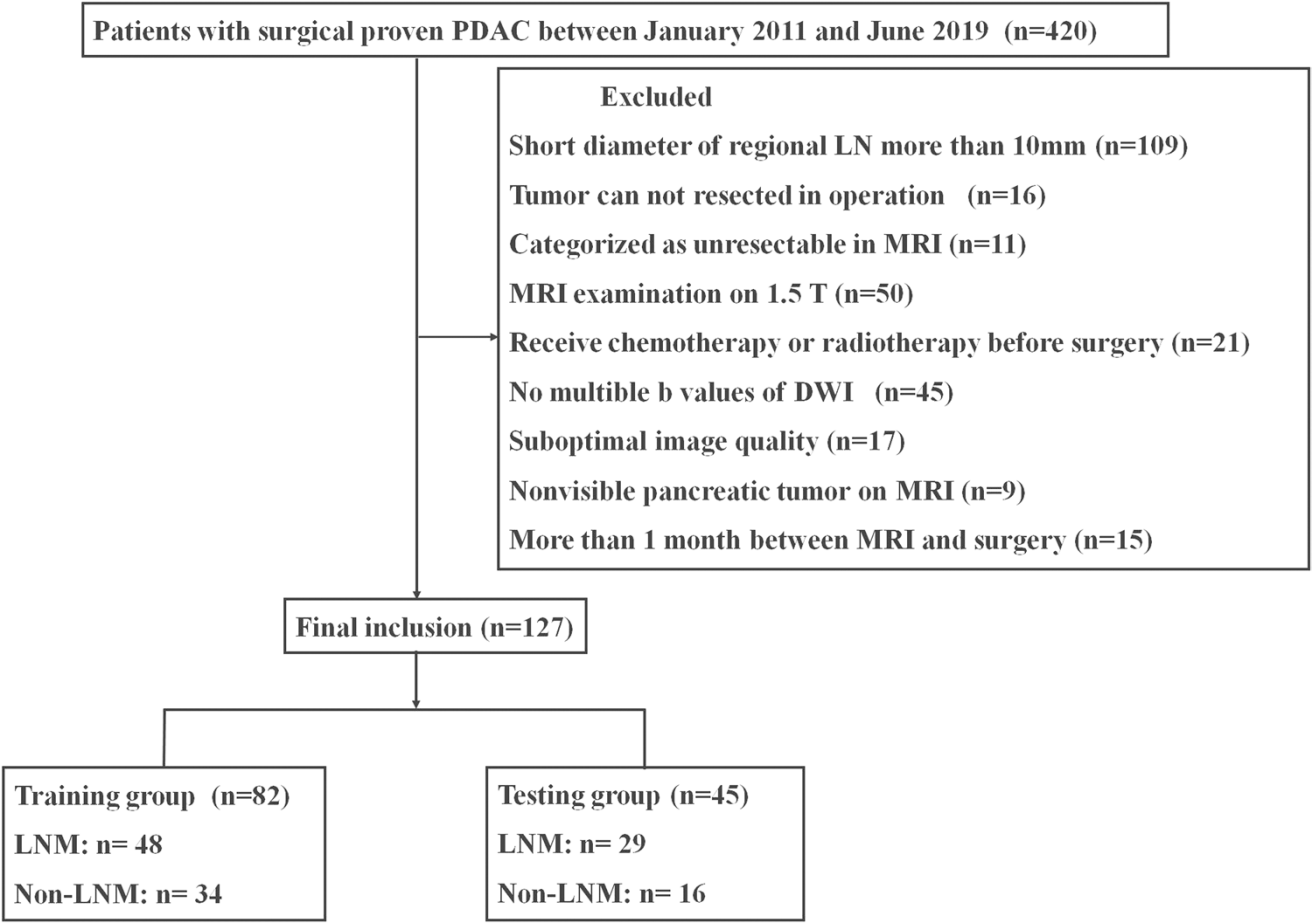
Patients flowchart

This study included patients who fulfilled the following inclusion criteria: (1) underwent preoperative 3 T pancreatic MRI; (2) MRI showed the pancreatic lesion was resectable or borderline resectable lesions according to NCCN criteria [14]; (3) underwent surgery and histological confirmation as PDACs; (4) their largest regional lymph node was <10 mm in short-axis diameter at pancreatic MR imaging; (5) did not receive chemotherapy or radiotherapy before surgery and MRI; (6) < 1 month between imaging and surgery; (7) had available diagnostic quality images for measuring lesions, without any severe motion of metallic artifacts. The regional lymph nodes in pancreatic head and uncinate cancer included lymph nodes along the common bile duct, common hepatic artery, portomesenteric vein and pancreaticoduodenal arcades. The regional lymph nodes in pancreatic body and tail cancer included lymph nodes along the common hepatic artery, celiac axis, splenic artery, and splenic hilum [15]. More remote nonregional lymph nodes, such as infrarenal or retroperitoneal lymph nodes in a paraaortic location or lymph nodes on the left of the superior mesenteric artery within the jejunal mesentery, equated to distant metastatic disease and was not included in our study [16]. A total of 127 consecutive patients with histopathologically diagnosed PDAC meeting inclusion criteria were included in the study between January 2011 and June 2019. Patients were allocated to the training and testing groups according to the time of surgery in a 2:1 ratio, where the first 82 patients were allocated to the training group, and the subsequent 45 patients were allocated to the testing group. The individual patients were regarded as the unit of analysis and classified as groups of lymph node metastases and non-lymph node metastases according to the pathologic diagnosis of lymph nodes. The complete patient enrollment process is shown in Fig. 1. The clinical characteristics of all patients are shown in Table 1. The study was approved by the institutional review board of our hospital. The requirement for informed consent was waived.

**Table 1:** Characteristics of the patients in the training and testing groups

Characteristics	Training group (n = 82)		*P*	Testing group (*n* = 45)		*P*
	Non-LNM (*n* = 34)	LNM (*n* = 48)		Non-LNM (*n* = 16)	LNM (*n* = 29)	
Clinical characteristics						
Age (years)	64.41 ± 11.24	64.58 ± 9.90	0.942	58.31 ± 10.24	62.86 ± 6.10	0.119
Sex, *n* (%)			0.086			0.577
Male	14 (41.2)	29 (60.4)		8 (50)	17 (58.6)	
Female	20 (58.8)	19 (39.6)		8 (50)	12 (41.4)	
CA 199, *n* (%)			0.978			0.296
Negative	7 (20.6)	10 (20.8)		6 (37.5)	6 (20.7)	
Positive	27 (79.4)	38 (79.2)		10 (62.5)	23 (79.3)	
CEA, *n* (%)			0.511			0.028
Negative	21 (61.8)	33 (68.8)		14 (87.5)	16 (55.2)	
Positive	13 (38.2)	15 (31.2)		2 (12.5)	13 (44.8)	
Qualitative analysis						
Location, *n* (%)			0.255			0.338
Head, uncinate	12 (35.3)	23 (47.9)		9 (56.3)	12 (41.4)	
Neck, body, tail	22 (64.7)	25 (52.1)		7 (43.7)	17 (58.6)	
Shape, *n* (%)			0.373			0.378
Diffuse	10 (29.4)	10 (26.3)		4 (25)	11 (37.9)	
Local	24 (70.6)	38 (73.7)		12 (75)	18 (62.1)	
Main stem of PV, SPV and SMV, *n* (%)			0.654			0.027
Non-invasion	16 (47.1)	25 (52.1)		11 (68.8)	9 (31.0)	
Invasion	18 (52.9)	23 (47.9)		5 (31.2)	20 (69.0)	
Branches of PV, SPV and SMV, *n* (%)			0.773			0.079
Non-invasion	5 (14.7)	6 (12.5)		5 (31.2)	2 (6.9)	
Invasion	29 (85.3)	42 (87.5)		11 (68.8)	27 (93.1)	
Main stem of GDA, SPA and SMA, *n* (%)			0.346			0.027
Non-invasion	18 (52.9)	23 (47.9)		11 (68.8)	10 (34.5)	
Invasion	16 (47.1)	25 (52.1)		5 (31.2)	19 (65.5)	
Branches of GDA, SPA and SMA, *n* (%)			0.773			0.111
Non-invasion	5 (14.7)	6 (12.5)		5 (31.2)	3 (10.3)	
Invasion	29 (85.3)	42 (87.5)		11 (68.8)	26 (89.7)	
Duodenum, *n* (%)			0.037			0.281
Non-invasion	32 (94.2)	37 (77.1)		16 (100.0)	25 (86.2)	
Invasion	2 (5.8)	11 (22.9)		0 (0)	4 (13.8)	
Bile duct, *n* (%)			0.428			0.726
Non-invasion	28 (82.4)	36 (75.0)		12 (75.0)	23 (79.3)	
Invasion	6 (17.6)	12 (25.0)		4 (25.0)	6 (20.7)	
Dilated MPD, *n* (%)			0.599			0.722
Yes	15 (44.1)	24 (50.0)		6 (37.5)	7 (24.1)	
No	19 (55.9)	24 (50.0)		10 (62.5)	22 (75.9)	
Cystic change, *n* (%)			0.062			0.256
Yes	18 (52.9)	35 (72.9)		10 (62.5)	13 (44.8)	
No	16 (47.1)	13 (27.1)		6 (37.5)	16 (55.2)	
Quantitative analysis						
Primary tumor						
Long axis (mm)	31.24 ± 12.66	29.48 ± 9.67	0.479	26.31 ± 15.86	30.10 ± 13.83	0.093
Short axis (mm)	19.97 ± 9.33	19.04 ± 5.83	0.581	16.69 ± 7.19	20.17 ± 6.86	0.116
ED of tumor (mm)	7.09 ± 6.40	12.90 ± 6.94	<0.001	5.13 ± 4.87	13.24 ± 5.31	<0.001
Distance to P (mm)	4.44 ± 4.32	4.35 ± 6.29	0.945	5.00 ± 4.53	1.76 ± 3.09	0.018
Largest LN						
Short diameter (mm)	4.74 ± 1.83	6.71 ± 1.75	<0.001	4.19 ± 1.87	6.55 ± 1.68	<0.001
DWI parameters						
ADC (× 10^–3^ mm^2^/s)	1.34 ± 0.31	1.20 ± 0.26	0.068	1.27 ± 0.29	1.16 ± 0.34	0.286
D (× 10^–3^ mm^2^/s)	1.02 ± 0.34	0.96 ± 0.23	0.408	1.02 ± 0.27	0.91 ± 0.26	0.794
D* (× 10^–2^ mm^2^/s)	8.16 ± 19.32	13.72 ± 47.41	0.977	13.61 ± 28.43	13.00 ± 25.71	0.393
f	0.38 ± 0.12	0.32 ± 0.10	0.024	0.42 ± 0.17	0.40 ± 0.17	0.589
DDC (× 10^–2^ mm^2^/s)	1.69 ± 0.44	1.43 ± 0.40	0.01	1.71 ± 0.71	1.60 ± 0.72	0.393
α	0.72 ± 0.11	0.71 ± 0.15	0.721	0.64 ± 0.20	0.67 ± 0.14	0.553
MD (× 10^–3^ mm^2^/s)	1.98 ± 0.35	1.69 ± 0.46	0.005	2.31 ± 0.74	1.73 ± 0.65	0.009
MK	0.75 ± 0.16	0.73 ± 0.22	0.829	0.73 ± 0.14	0.69 ± 0.25	0.759

*D* Diffusion, D* Perfusion, *α* Diffusion heterogeneity index, *DDC* Distributed diffusion coefficient, *ED* Extrapancreatic distance, *f* Fraction, *GDA* Gastroduodenal artery, *n* Number, *LN* Lymph node, *LNM* Lymph node metastasis, *MD* Mean diffusivity, *MK* Mean kurtosis, *MPD* Main pancreatic duct, *P* Peritoneum, *PDAC* Pancreatic ductal adenocarcinoma, *PV* Portal vein, *SMA* Superior mesenteric artery, *SMV* Superior mesenteric vein, *SPA* Splenic artery; *SPV* Splenic vein

Data are presented as mean ± standard deviation

### MR imaging technique

MR examinations were performed on a 3 T MR system (Discovery 750; GE Healthcare, Waukesha, WI, USA) with 8-channel phased-array receiver coils in the supine position. The MR imaging protocol consisted of routine pancreatic imaging sequences and DW imaging sequences. The conventional MRI protocols included the following sequence: (1) a respiration-triggered axial T2-weighted fast spin-echo (FSE) (TR/TE, 8000/109 ms; matrix, 288 × 256; NEX, 4; slice thickness/gap, 5/1 mm); (2) pre-contrast imaging including fat-suppressed (FS) T1-weighted imaging were obtained with a 3D lava-flex sequence in one breath-hold (TR/TE, 3.2/2 ms; matrix, 256 × 192; NEX, 1; slice thickness/gap, 5/−2.5 mm); (3) dynamic contrast-enhanced MRI was acquired using lava-flex sequence (TR/TE, 3.2/2 ms; matrix, 256 × 192; NEX, 1; slice thickness/gap, 5/−2.5 mm); arterial, portal vein and delayed phase imaging were obtained approximately 25 s, 60 s and 2 min, respectively, after the start of contrast material administration. Intravenous injection of gadolinium-diethylenetriamine pentaacetic acid (DTPA) (Magnevist; Bayer Schering, Berlin, Germany) at 0.1 mmol/kilogram of body weight and flow rate 2 ml/s was used, followed by a 15-ml saline flush.

DWI was performed before enhanced imaging. Axial DWI was acquired by a respiratory triggered free-breathing single-shot echo-planar imaging using the navigator echo technique. The sequence parameters were as follows: TR/TE, 7000/60 ms; slice thickness/gap, 3/0.5 mm; the number of gradient directions of 3; FOV of 390 × 310 mm, the bandwidth of ± 250 kHz; flip angle of 90°; the integrated parallel acquisition techniques imaging option with a factor of 3; fat suppression technique; distortion correction technique to avoid artifacts; the NEX of b = 0–800, 1000, 1200, and 1500 s/mm^2^ were 1, 2, 4, and 6, respectively; a total of 10 b values (0, 20, 50, 100, 200, 600, 800, 1000, 1200, and 1500 s/mm^2^).

### Image analysis

All images were reviewed using a local picture archiving and communication system. The reviewers were blinded to the clinical data, imaging results, and final diagnosis of lymph nodes; however, they were aware that the study population was PDAC. All MR images were independently analyzed and recorded by two abdominal radiologists (Dr. SS Sun and Dr. B Zhao with 6 and 4 years of experience reading abdominal MR images). Quantitative variables were recorded as the average of two separately measurement by two radiologists. For qualitative analysis, any discrepancy during analysis was resolved through achieving consensus by consulting a senior abdominal radiologist (Dr. Shi, 12 years of experience reading body MRI).

#### Qualitative analysis

We analyzed all patients’ data for demographic characteristics including sex, age, and laboratory examinations (CA 199 and CEA). Tumor-specific variables including location, characteristics of pancreatic cancer, and presence of main pancreatic duct (MPD) dilatation, arterial and venous invasion, duodenal invasion, and bile duct invasion were evaluated.

Tumor location was categorized into two groups; the first group, in which the tumor was located in the head or uncinate of the pancreas; the second group, in which the tumor was located in the neck, body, or tail of the pancreas. Characteristics of PDACs contained the presence of cystic components and morphologic patterns. Cystic components were defined as markedly high signal intensity areas on T2WI and displayed no enhancement in any dynamic phase. The morphologic patterns of tumors were graded as focal and diffuse type. Dilatation of MPD was defined as MPD diameter at the tail side was dilated compared with the MPD diameter at the ampullary side. Imaging findings related to vessel invasion in PDACs were classified as negative and positive. Peripancreatic vessels of unilateral or bilateral narrowing and stenosis or obstruction with collaterals were considered as vessel invasion. The observed vessels contained portal vein, gastroduodenal artery, splenic artery and vein, superior mesenteric artery and vein, and their branches.

We attempted to track the regional LNs by using LN size, signal intensity, margin, as well as its distance from the tumor [15, 17, 18]. A hyperintensity lymph node on high b values of DWI, or presence of internal necrosis in LNs, or irregular margin of LNs, or LNs near the tumor was diagnosed as positive for metastases. The short axis of LN was interpreted as follows: the larger the short-axis diameter of the lymph node, the higher the probability of metastasis. When one or more lymph nodes were diagnosing as metastatic LN in an individual patient, this patient was categorized as LNM group. When no lymph nodes was diagnosing as metastatic LN in an individual patient, this patient was categorized as non-LNM metastasis group.

#### Quantitative analysis

**Fig. 2:**
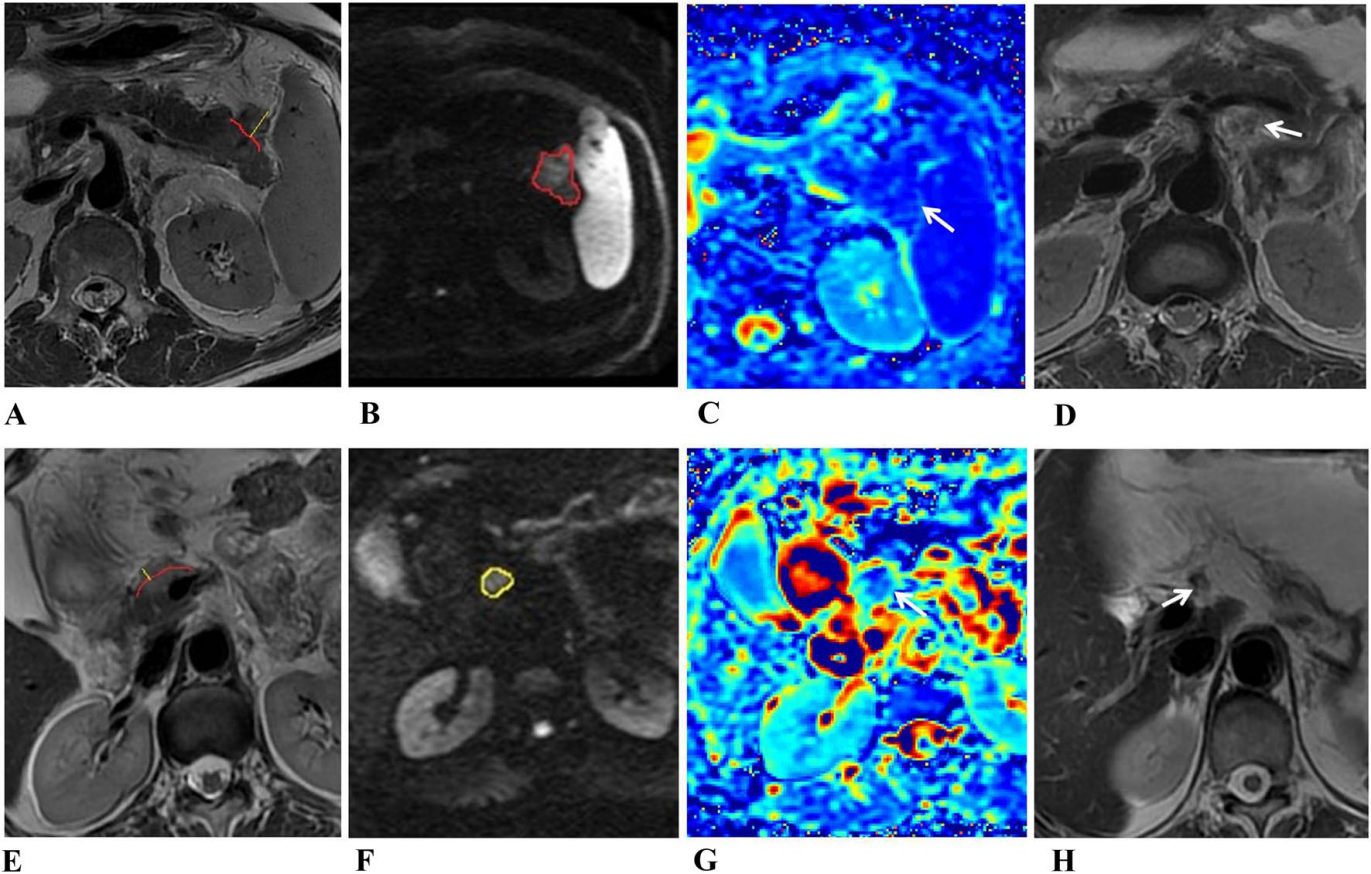
(**a–d**) MR images of a 58-year-old man with pancreatic carcinoma with positive LNM. **a** Axial T2-weighted image showed an irregular high signal intensity tumor in the tail of the pancreas; the extrapancreatic distance (yellow line) of tumor invasion was 17 mm. **b** DWI with b = 1500 s/mm^2^ showed a hyperintense tumor; the ROI was drawn, including the entire tumor. **c** On the MD parametric map, the tumor showed an isointense signal with a value of 1.54 × 10^–3^ mm^2^/s, which was less than the cutoff value of 1.74 × 10^–3^ mm^2^/s. **d** On axial T2-weighted image, diameter of LN around the splenic artery was 7 mm (arrow). **e–h** MR images in a 56-year-old woman of pancreatic carcinoma with negative LNM. **e** Axial T2-weighted image showed a well-defined high signal intensity tumor in the head of the pancreas; the extrapancreatic distance (yellow line) of tumor invasion was 6 mm. **f** DWI with b = 1500 s/mm^2^ showed a hyperintense tumor; the ROI was drawn, including the entire tumor. **g** On the MD parametric map, the tumor showed an isointense signal with a value of 2.70 × 10^–3^ mm^2^/s. **h** Axial T2-weighted image showed LN with a diameter of 5 mm around the pancreatic head

The diameters of the short and long axis of tumors were measured on the T2WI sequence. Extrapancreatic distance of tumor invasion was determined by measuring the distance between the outer border of the pancreas and the outermost border of the tumor on the T2WI sequence. Junctions between tumor and normal parenchyma were identified and then according the outline of normal pancreas we drew outer border of the pancreas between the two junctions. While the tumor was located in the body or tail of the pancreas, the outer border of the pancreas may be straight; when the tumor was located in the head or uncinate, the outer border of the pancreas may be curved (Fig. 2). The radiologists measured the shortest distance between peritoneum and tumor, and the short-axis diameter of the largest lymph node. The largest lymph node was used for quantitative evaluation if two or more positive nodes were observed in MRI.

The ROIs were manually drawn with the ITK-SNAP software (version 3.8.0) using DWI with a b value of 1500 s/mm^2^ on each slice. The tumor was contoured slice by slice to obtain the entire neoplastic ROIs. The ROIs were placed on the tumor region, as determined by T1WI, T2WI, DWI, and contrast imaging. Special attention was taken to avoid normal pancreatic tissue, pancreatitis, or adjacent normal vasculature. Parameters of DWI were obtained through the ROIs.

#### Parameter estimation

After ROI delineation, the parameters of the non-gaussian DWI models were calculated. The IVIM parameters contained D, D* and f. D was the slow component of diffusion reflecting pure molecular diffusion, D* was the fast component of diffusion associated with perfusion and representing incoherent microcirculation, and f was the volume fraction of the protons linked to the intravascular component or to the microcirculation [19]. The SEM parameters included DDC (distributed diffusion coefficient) and α. DDC represented the mean intravoxel diffusion rate, and α was the intravoxel water molecular diffusion heterogeneity index, which characterized the deviation of signal attenuation from monoexponential behavior and ranged from 0 to 1. A value of α near 1 indicated high homogeneity in apparent diffusion [20]. DKI parameters contained D_app_ and K_app._ D_app_ was the apparent diffusion coefficient (in mm^2^/s), and K_app_ was the apparent diffusion kurtosis coefficient [21].

### Surgery and histopathology

Pancreatic tumor surgery with regional LN dissection is the standard treatment procedure for patients with PDACs at our hospital. Pancreaticoduodenectomy (*n* = 56) and distal pancreatectomy (*n* = 71) were chosen according to tumor location. The surgeons were able to accurately label all specimens. In particular, regional lymph node specimens were dissected according to the NCCN criteria. The lymph nodes were dissected from the specimen, and nodes were examined separately. The lymph nodes metastasis showed ill-defined, round and slightly hard texture lesion with internal gray and white tissue containing area of necrosis in gross specimen. However, it was very difficult to differentiate LNM and non-LNM in gross specimen sometimes. One pathologist with a special interest in PDAC examined all pathologic specimens. Pathological slides were stained with hematoxylin and eosin and analyzed by light microscopy. Lymph nodes metastasis was confirmed by presence of the pancreatic cancer cell in the lymph nodes. Non-lymph nodes metastasis was diagnosed by absence of tumor cell and presence of leukomonocyte and lymphatic sinusoid. The presence of microscopic tumor invasion into the adjacent organ, and vessel invasion were determined. The results of the analysis of the surgical specimen were used as a reference standard.

### Statistical analysis

The continuous variables were analyzed using Student’s t-test or Mann–Whitney U test. Categorical variables were analyzed using the Chi-square test. Multivariate analysis of factors predicting LNM was performed using logistic regression. Variables with *P* < 0.05 in univariate analysis were incorporated into a multivariate logistic regression model. The receiver operating characteristic (ROC) curve was used to assess the diagnostic performance. The area under the curve (AUC), sensitivity, specificity, and accuracy were calculated. The AUCs between the MRI model and radiologists were compared using the z-test. The interobserver agreement between the two radiologists was evaluated using kappa statistics. Overall survival was calculated from 1 January 2011 until cancer-specific death. Follow-up assessment consisted of outpatient interviews at 3-month intervals for 2 years, then at 6-month intervals for 3 years, and finally at 12-month intervals until death. No patient was lost to follow-up. Kaplan–Meier method with log-rank test was conducted to compare survival curves between LNM and non-LNM groups. All analyses were conducted using SPSS 22.0 (IBM Corporation, Armonk, NY, USA) and STATA 12.0 (Stata Corporation, College Station, TX, USA).

## Results

### Characteristics of patients

There were 127 eligible patients with pathologically proven PDACs, 59 women and 68 men aged 36–88 years, with mean age of 63.6 ± 9.6 years. The characteristics of the patients are summarized in Table 1. The kappa values of subjective diagnosing of LNM in PDACs for the two independent radiologists were 0.43, which indicated moderate agreement. The interobserver agreements of the two radiologists for measuring parameters of the DWI models, which calculated based on the ROIs of tumor, achieved a satisfactory agreement with a kappa value of 0.85. The interobserver agreements of the two radiologists for measurement of extrapancreatic distance in tumor and the short-axis diameter of the largest lymph node were substantial with kappa values of 0.77 and 0.74, respectively. Two radiologists qualitatively assessed the PDACs with kappa values of 0.70–0.78, indicating substantial agreement.

### Univariable comparisons of quantitative and qualitative analysis

Table 1 shows the univariable comparisons of qualitative and quantitative analysis using MRI for predicting the lymph node metastases. Extrapancreatic distance of tumor invasion, short-axis diameter of the largest lymph node and mean diffusivity (MD) of tumor significantly differed between negative and positive lymph node metastases groups. Extrapancreatic distance of tumor invasion and short-axis diameter of the largest lymph node of the positive LNM group were significantly larger compared to the negative LNM group. The MD of tumors in the positive LNM group was significantly lower compared to the negative LNM group. All these characteristics were included in the LNM evaluation model (Fig. 2).

### Diagnostic performance of MRI model for predicting lymph node metastases

Table 2 shows the adjusted logistic regression models for diagnosing metastatic and non-metastatic lymph nodes. We found that the short-axis diameter of the largest lymph node had the highest AUC value (0.784), followed by that of the extrapancreatic distance of tumor invasion (0.729) and MD (0.681). This analysis revealed that for differentiating between metastatic and non-metastatic lymph nodes, the short-axis diameter of the largest lymph node had a higher rate of diagnosing metastatic lymph nodes [odds ratio (OR) = 1.528; 95% confidence interval (CI): 1.104–2.113]. Short-axis diameter of the largest lymph node with a cutoff value of larger than 5.5 mm, ED of tumor invasion with a cutoff value of larger than 13.5 mm, and MD with cutoff values of less than 1.74 × 10^–3^ mm^2^/s to predicting LNM in PDACs yielded the accuracy of 74.4%, 65.9%, and 70.7%, respectively.

**Table 2:** Multivariable logistic regression results of parameters obtained from MRI model for predicting lymph node metastases

Measurement	B	OR	95% CI	*P*
ED of tumor invasion	0.099	1.104	1.015–1.201	0.021
Short diameter of largest LN	0.424	1.528	1.104–2.113	0.011
MD (×10^3^ mm^2^/s)	−1.638	0.194	0.039–0.979	0.047

*B* Regression coefficient, *CI* Confidence interval, *ED* Extrapancreatic distance, *MD* Mean diffusivity, *OR* Odds ratio

**Fig. 3:**
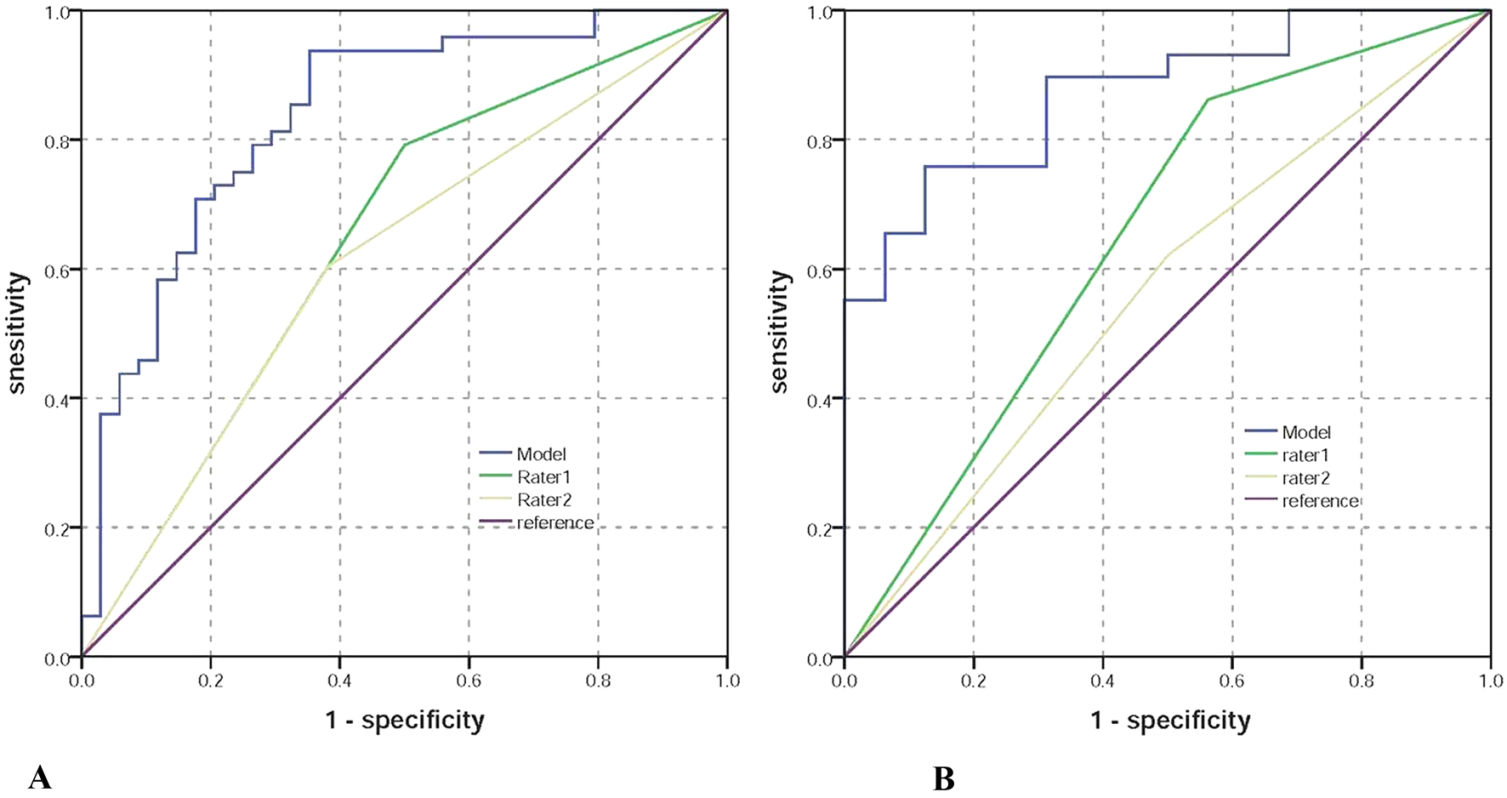
Receiver operating characteristics (ROC) curves analysis of the MRI model and subjective diagnosis for predicting small LNM in the training group (**a**) and testing group (**b**). The blue line presents the performance of the MRI model, the green and yellow lines present the performance of rater 1 and rater 2, and the purple line the reference. **a** The AUCs of the MRI model, rater 1 and rater 2 for predicting small LNM, were 0.836, 0.646, and 0.611 in the training group. **b** The AUCs of MRI model, rater 1 and rater 2 were 0.873, 0.633, and 0.560 for predicting small LNM were in the testing group

According to the results of the logistic regression model, extrapancreatic distance of tumor invasion, short-axis diameter of the largest lymph node, and MD of tumor were used to establish a diagnostic model to predict metastatic lymph nodes. A combined MRI model was established using the following formula: value = 0.099 × extrapancreatic distance of tumor invasion + 0.424 × short-axis diameter of the largest lymph node − 1.638 × MD × (10^3^). The patient with value from combined MRI model larger than the cutoff value of −0.33 was classified as LNM group. The combined MRI diagnostic model yielded an AUC of 0.836 and an accuracy of 81.7% in the training group and an AUC of 0.873 and an accuracy of 80% in the testing group (Fig. 3). Detailed information on the performance of the combined MRI model for predicting metastatic lymph nodes is shown in Table 3.

**Table 3:** Performance of combination of MRI model and subjective diagnosis for predicting LN in PDACs

		AUC	SEN (%)	SPE (%)	PPV (%)	NPV (%)	ACU (%)	Error rate (%)
Training group	MRI model	0.836 (0.746–0.927)	93.8	64.7	78.9	88	81.7	18.3
	Rater 1	0.646 (0.522–0.770)	79.2	50	69.1	63	67.1	32.9
	Rater 2	0.611 (0.487–0.735)	60.4	61.8	69	52.5	61	39
Testing group	MRI model	0.873 (0.773–0.973)	75.6	87.5	91.7	66.7	80	20
	Rater 1	0.633 (0.455–0.810)	82.8	43.8	72.7	58.3	68.9	31.1
	Rater 2	0.560 (0.383–0.738)	62.1	50	69.2	47.1	57.8	42.2

*AUC* Area under curve, *SEN* Sensitivity, *SPE* Specificity, *ACU* Accuracy

### Comparison between the subjective evaluation and the MRI model for predicting lymph node metastases

**Fig. 4:**
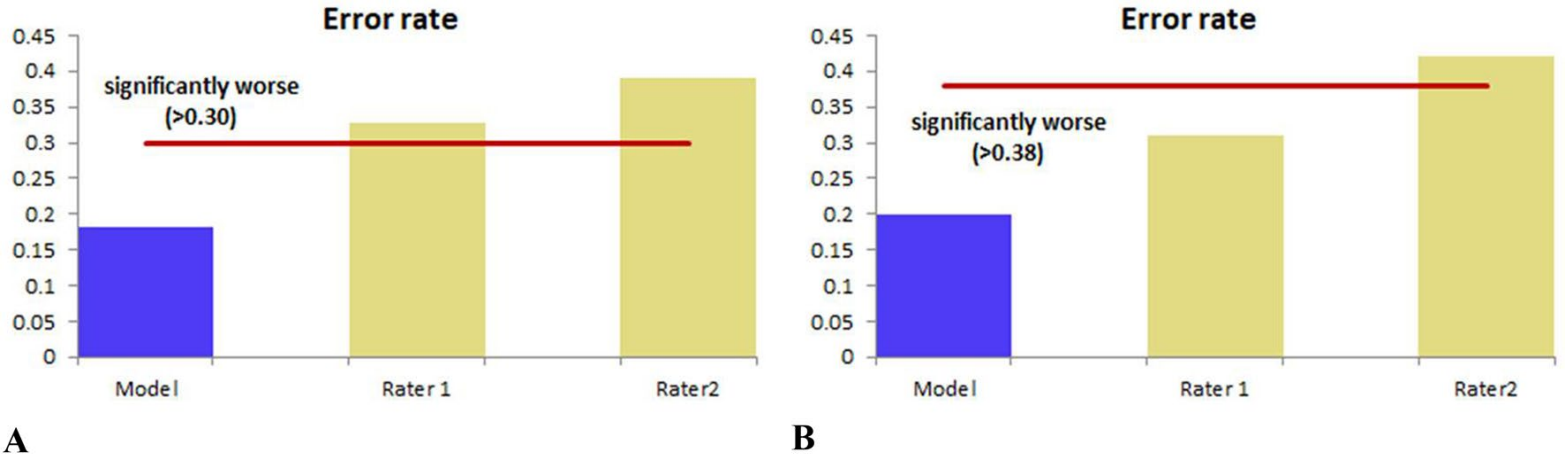
Bar graphs showing the comparison of the error rates among subjective evaluation and the MRI model for predicting small LNM for patients with PADCs in the training group (**a**) and testing group (**b**). A lower error rate indicates a better performance. **a** The error rate of the subjective evaluation for predicting small LNM was higher than that of MRI model in the primary group (both *P* < 0.05). **b** The error rate of the subjective evaluation for predicting small LNM was higher than that of the MRI model in the testing group (Rater 1, *P* > 0.05; Rater 2, *P* < 0.05)

The sensitivity, specificity, positive predictive value, negative predictive value, and diagnostic accuracy of subjective MRI diagnosis for lymph nodes metastases reported by two radiologists are summarized in Table 3. The AUC of the MRI model for predicting MLN was higher than that of subjective MRI diagnosis in the training group (*P* = 0.01 and 0.008) and in the testing group (*P* = 0.036 and 0.024). Comparing the subjective diagnosis, the error rate of the MRI model was decreased (Table 3, Fig. 4).

### Survival analysis between model-defined and pathologic lymph node metastases groups

**Fig. 5:**
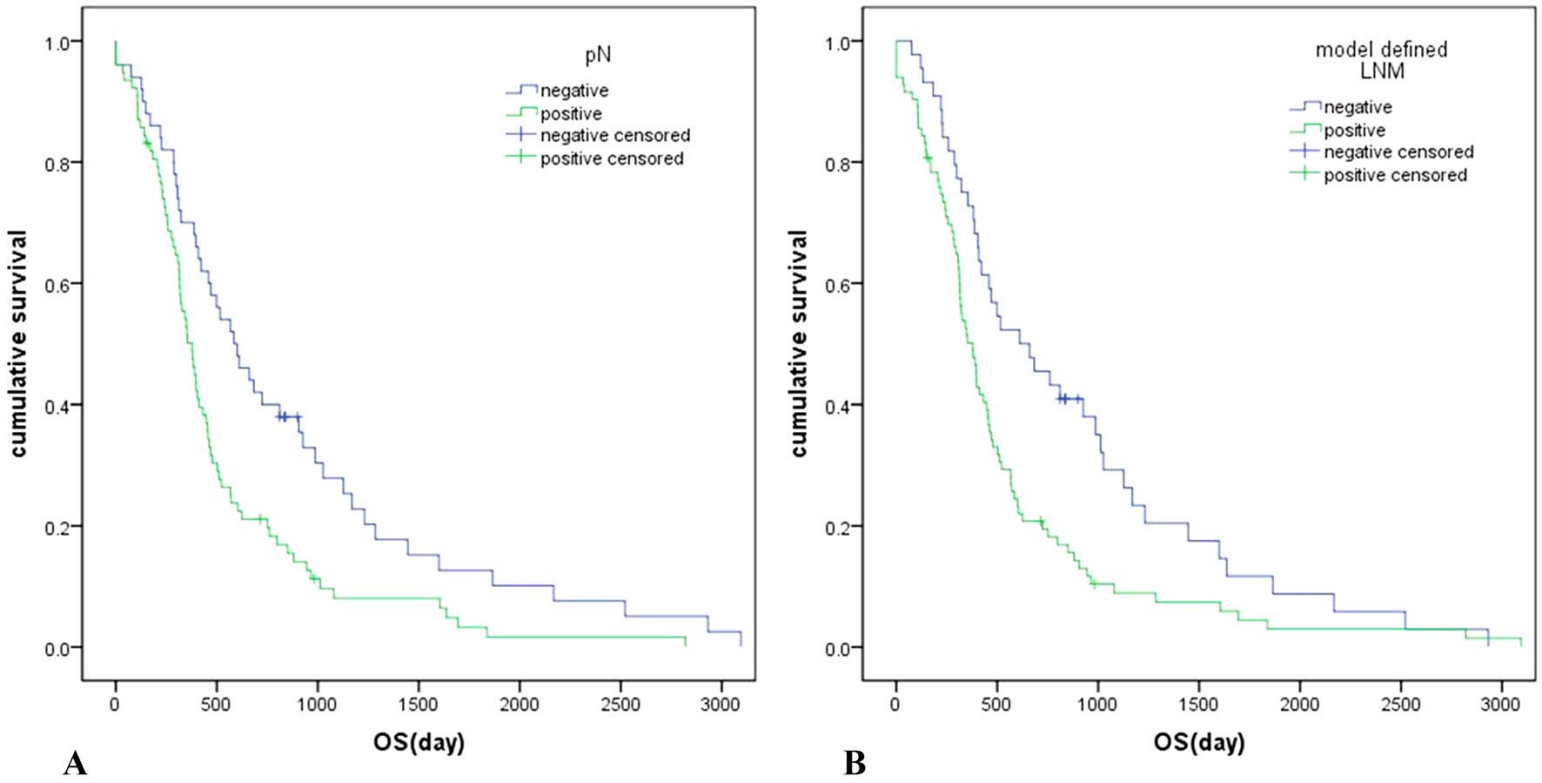
Kaplan–Meier curves after resection of pancreatic cancer. **a** Association between pathological lymph node status and survival outcomes. **b** Association between lymph node status of MRI model and survival outcomes

A total of 120 patients (94.5%) died, and the mean follow-up data was 408 days (95% CI, 308 to 494 days). The model-defined LNM-positive group showed significantly inferior overall survival than the negative group (median survival: 377 days vs. 612 days, *P* = 0.006). The pathological LNM-positive group also showed inferior overall survival than the negative group (median survival: 377 days vs. 584 days, *P* = 0.003) (Fig. 5).

## Discussion

Preoperative prediction of small LN metastases in patients with PDACs is a very important challenge to overcome. Previous reports provided strong support for preoperative chemotherapy for patients with node-positive pancreatic cancer, one-third of whom may be downstaged; 5 year OS for this group was 27.2% vs. 12.3% for N1 patients who only received surgery [22]. Nodal downstaging was associated with a survival benefit in patients with node-positive PDAC [23]. The traditional criteria for diagnosing LNM were a short diameter of LN more than 10 mm, which may have relatively low sensitivity and be omitted for small lymph node metastases [23, 24]. In addition, previous studies highlighted the difficulty of identifying patients at high risk of LNM solely by assessing tumor size [10, 25]. Due to the lack of sufficient preoperative predictors of LN metastases, the optimal indication for selecting patients accepting preoperative chemotherapy has not yet been established. Therefore, in the present study, we established a MRI model to improve the diagnostic performance for small lymph nodes in PDACs.

Our results revealed that a short diameter with more than 5.5 mm of lymph node was one of the independent predictor for predicting small LNM. Some previous studies also found that reducing the size threshold for characterizing LNM enhanced the sensitivity [12, 24, 26]. In clinical practice, the criteria of metastatic lymph nodes was vague and the performance of differentiating the malignant lymph nodes from the benign ones based on morphologic features by radiologists mainly based on clinical experience was limited. In this study, the AUCs of subjective diagnosis of metastatic lymph nodes in PDACs were 0.56–0.65 and the interobserver agreement was moderate. We choose to measure the largest lymph node instead of the most suspicious metastatic lymph node. Identifying most suspicious metastatic lymph node was subjective and reproducibility may be low. However, largest lymph node around the pancreas was easily observed in MRI and substantial interobserver agreement with kappa value of 0.74 showed that reproducibility of clinical use was well. Reducing the size threshold for characterizing LNM enhanced the sensitivity of diagnosing LNM. Despite the loss of specificity, the performance of diagnosing LNM using threshold value of short axis less than 10 mm was improved.

We found that extrapancreatic distance of tumor invasion was identified as an independent prognostic factor for LNM. Lymphatic vessels and blood vessels located around the pancreas were tubular structures surrounded by endothelial cells. Postcapillary venules and lymphatic vessels were both thin-walled and small-caliber vessels [27]. We supposed that extrapancreatic invasion might be correlated with the invasion of lymphatic vessels, thus increasing the possibility of LNM. Our result also showed that this parameter measurement resulted in the substantial interobserver agreement, which indicated that high repeatability and reproducibility of this parameter could be achieved in clinical practice.

DKI is an extension of DWI that evaluates the microstructure features of tissues in a non-Gaussian model [28]. MD reflecting diffusion was also correlated with tissue microstructure, such as cell density and nucleus. When cytoplasm ratio or cell density increased, the extracellular space was reduced, the diffusion movement of water molecules was limited, and the MD value was decreased [28]. The aggressive biological behavior of tumors may be associated with the difference in tumor microstructure. Our results suggested that MD of the tumor with LNM had a lower MD value, which is consistent with most previous studies on pancreatic cancer and cholangiocarcinoma [13, 24, 28].

In this study, the MRI model for predicting small LNM increased the diagnostic performance compared to the short diameter of largest LN alone. The performance of this MRI model for predicting LNM was higher than that of subjective MRI diagnosis. Hence, this MRI model could improve the performance and confidence of radiologists in predicting LNM and assist doctors in accurately choosing appropriate management. The MRI model defining LNM-positive group showed significantly inferior OS than the negative group. This finding confirmed the excellent performance of this MRI model for predicting small LNM in patients with pancreatic carcinoma.

Our study has several limitations. First, this was a retrospective study that was performed at a single center; the number of included patients was small, mainly because of the strict inclusion criteria; a large sample study from multicenter is needed to validate this reported findings. Second, there might be selection bias for enlarged LN because we did not perform radiological–pathological 1–1 matching on a per-LN basis using the direction and LN size; some small lymph nodes with microscopic metastatic foci were beyond the scope of MRI. Third, we did not assess the DWI feature of LN, as small lymph nodes may lead to measurement bias when drawing the ROI of LN. Fourth, locally advanced PDACs with regional metastatic lymph node of short-axis diameter larger than 10 mm may not be suit to apply this model. Fifth, when the PDAC extended into the peritumoral space or lymph nodes did not be separated from PDAC, it was difficult to differentiate peritumoral lymph nodes and tumor extrapancreatic invasion in MRI, and this condition may be limited to apply this model in clinical practice. The further evaluation of N staging of PDACs should focus on searching the other morphological and functional features in MRI by performing radiological–pathological 1–1 matching. To explore the value of MRI for evaluating the preoperative staging and treatment response of metastatic lymph nodes in PDACs after neoadjuvant chemotherapy is another direction in the future research.

In conclusion, we established a MRI model with excellent performance for individualized and noninvasive prediction of small lymph nodes metastases in PDACs. Therefore, utilization of this MRI model for predicting LNM in patients with PDACs may improve the diagnostic accuracy and help to establish timely and appropriate treatment.

## Abbreviations

ADC: Apparent diffusion coefficient
AUC: Area under the curve
DKI: Diffusion kurtosis imaging
DWI: Diffusion-weighted imaging
DDC: Distributed diffusion coefficient
ED: Extrapancreatic distance
IVIM: Intravoxel incoherent motion
LN: Lymph node
LNM: Lymph node metastases
MD: Mean diffusivity
MK: Mean kurtosis
MLN: Metastatic lymph node
MPD: Main pancreatic duct
MRI: Magnetic resonance imaging
PDACs: Pancreatic ductal adenocarcinomas
ROC: Receiver operating characteristic
SEM: Stretched exponential model
